# Antileishmanial High-Throughput Drug Screening Reveals Drug Candidates with New Scaffolds

**DOI:** 10.1371/journal.pntd.0000675

**Published:** 2010-05-04

**Authors:** Jair L. Siqueira-Neto, Ok-Ryul Song, Hyunrim Oh, Jeong-Hun Sohn, Gyongseon Yang, Jiyoun Nam, Jiyeon Jang, Jonathan Cechetto, Chang Bok Lee, Seunghyun Moon, Auguste Genovesio, Eric Chatelain, Thierry Christophe, Lucio H. Freitas-Junior

**Affiliations:** 1 Center for Neglected Diseases Drug Discovery (CND3), Institut Pasteur Korea, Seongnam-si, Gyeonggi-do, South Korea; 2 Screening Technology & Pharmacology Group, Institut Pasteur Korea, Seongnam-si, Gyeonggi-do, South Korea; 3 Active Compound Space Group, Institut Pasteur Korea, Seongnam-si, Gyeonggi-do, South Korea; 4 Image Mining Group, Institut Pasteur Korea, Seongnam-si, Gyeonggi-do, South Korea; 5 Drugs for Neglected Diseases initiative (DNDi), Geneva, Switzerland; AP-HP, service de parasitologie-mycologie, France

## Abstract

Drugs currently available for leishmaniasis treatment often show parasite resistance, highly toxic side effects and prohibitive costs commonly incompatible with patients from the tropical endemic countries. In this sense, there is an urgent need for new drugs as a treatment solution for this neglected disease. Here we show the development and implementation of an automated high-throughput viability screening assay for the discovery of new drugs against *Leishmania*. Assay validation was done with *Leishmania* promastigote forms, including the screening of 4,000 compounds with known pharmacological properties. In an attempt to find new compounds with leishmanicidal properties, 26,500 structurally diverse chemical compounds were screened. A cut-off of 70% growth inhibition in the primary screening led to the identification of 567 active compounds. Cellular toxicity and selectivity were responsible for the exclusion of 78% of the pre-selected compounds. The activity of the remaining 124 compounds was confirmed against the intramacrophagic amastigote form of the parasite. *In vitro* microsomal stability and cytochrome P450 (CYP) inhibition of the two most active compounds from this screening effort were assessed to obtain preliminary information on their metabolism in the host. The HTS approach employed here resulted in the discovery of two new antileishmanial compounds, bringing promising candidates to the leishmaniasis drug discovery pipeline.

## Author Summary

Every year, more than 2 million people worldwide suffer from leishmaniasis, a neglected tropical disease present in 88 countries. The disease is caused by the single-celled protozoan parasite species of the genus *Leishmania*, which is transmitted to humans by the bite of the sandfly. The disease manifests itself in a broad range of symptoms, and its most virulent form, named visceral leishmaniasis, is lethal if not treated. Most of the few available treatments for leishmaniasis were developed decades ago and are often toxic, sometimes even leading to the patient's death. Furthermore, the parasite is developing resistance to available drugs, making the discovery and development of new antileishmanials an urgent need. To tackle this problem, the authors of this study employed the use of high-throughput technologies to screen a large library of small, synthetic molecules for their ability to interfere with the viability of *Leishmania* parasites. This study resulted in the discovery of two novel compounds with leishmanicidal properties and promising drug-like properties, bringing new candidates to the leishmaniasis drug discovery pipeline.

## Introduction

Leishmaniasis is a neglected emerging disease without any adequate treatment adapted to the field [1]. The disease can be characterized by skin ulcers (cutaneous leishmaniasis), mucous degeneration, especially from the mouth and internal nose (mucocutaneous leishmaniasis), and visceral organ damage (visceral leishmaniasis), which is lethal if untreated. The different forms of leishmaniasis manifestation depend mainly on the species of parasite but are also related to the host immune system. Official World Health Organization (WHO) numbers from the 1990s are still used and estimate 12 million infected people and 350 million at risk living in one of the 88 endemic countries in America, Europe, Africa, the Middle East and Asia [2]. The number of deaths as a consequence of leishmaniasis is higher than 50,000 per year, with an incidence of 1.5 million annual registered cases of the disfiguring cutaneous leishmaniasis and 0.5 million annual registered cases of the potentially fatal visceral leishmaniasis [3], but these numbers probably underestimate the real burden of the disease [4],[5].

Leishmaniasis is caused by the kinetoplastid species from the genus *Leishmania*. Infection takes place when a sandfly vector inoculates *Leishmania* promastigotes into the mammalian bloodstream; these extracellular flagellated forms of the parasite live in the insect midgut. Once in the bloodstream, parasites are phagocytosed by mononuclear blood cells, especially macrophages, differentiating into the obligatory intracellular amastigote form. Amastigotes proliferate inside the macrophages before inducing the bursting of the host cell and being released into the bloodstream. This process occurs repeatedly, leading to tissue damage [6]. Parasite species and the host immune system determine the clinical status of the disease, ranging from cutaneous ulcers (cutaneous leishmaniasis) [7] to visceral organ damage (visceral leishmaniasis) [8], especially of the spleen and the liver. Most of the antileishmanial drugs currently in use for treatment, from the long time established antimonials to the recently introduced miltefosine, have disadvantages, such as patient toxicity, side effects and/or parasite resistance [9].

Lead discovery is currently one of the bottlenecks in the pipeline for novel antileishmanial drugs [10]. High-throughput screening (HTS) optimizes the chance of finding lead compounds through the identification of active compounds from a large number of candidates [11]–[12]. We adapted an *in vitro* fluorometric assay to HTS format using the promastigote form of *L. major* [13], one of the causative species of cutaneous leishmaniasis. This was the first reference strain used to sequence the genome of this parasite, completed in 2005 [14], and genome information can be accessible for future studies, including target identification or mechanism of action determination. To validate the assay in HTS format, we screened a 4,000-compound library containing many bioactive compounds with known pharmacological properties, including currently used antileishmanials. Following validation, the assay was applied to the screening of a library containing 26,500 structurally diverse chemical compounds. A total of 567 compounds showing a minimum of 70% growth inhibition of the parasite (*L. major*) were identified during the primary screening at 10 µM. Further tests on their cytotoxicity on a human macrophage cell line and specificity filtering were applied, resulting in a list of 124 active compounds. To confirm activity against the intracellular parasites, these 124 compounds were tested in serial dilutions against *L. major* amastigotes infecting THP-1 differentiated macrophages. Through this process, the two most active compounds with EC_50_ values lower than 10 µM against *L. major* were chosen for further characterization. The 124 active compounds were also tested against intramacrophagic *L. donovani*, one of the causative species of visceral leishmaniasis, to evaluate specificity of the compounds against the parasites causing different clinical manifestations of the disease. To determine the quality of the two most active compounds, we tested their microsomal stability, which would indicate the presence of metabolites, as well as cytochrome P450 (CYP) inhibition for drug-drug interaction in multitherapies. The results indicate that the compounds are good candidates for further characterization for leishmaniasis therapy. We discuss the relevance of a developed HTS assay using the promastigote form of the parasite for the discovery of leishmanicidal compounds and the potential of one of the selected hits to become a future lead compound against leishmaniasis.

## Methods

### Parasites

*L. major* MHOM/IL/81/FRIEDLIN and *L. donovani* MHOM/ET/67/HU3 promastigotes were cultivated at 28°C in axenic M199 culture medium (Welgene, S. Korea) supplemented with 10% heat-inactivated fetal bovine serum (FBS) (Gibco, United States) and 1% streptomycin/penicillin (Gibco, United States).

### Compound Libraries, Reference Compound and Assay Plate Preparation

A total of 4,000 small molecules sourced from Sigma, Prestwick and Tocris were all screened at 2–20 µM, 0.2–2 µM and 0.02–0.2 µM. The 26,500-compound library screened at 10 µM (in 1% DMSO) was sourced from TimTec. This small molecule library contains compounds selected for diversity and drug-like properties as well as small collections of kinase-focused and protease-focused compounds. Ethidium bromide (EtBr) (Sigma E1510, United States), amphotericin B (Sigma A9528, United States), miltefosine (A.G. Scientific H-1096, United States) and paromomycin sulfate salt (Sigma P9297, United States) were used as reference compounds.

### Primary Screening Assay: Antileishmanial Activity

After compound addition to the assay plate (Evotec™ 384-well microplate, Germany), 20,000 *L. major* promastigote parasites from an exponential phase culture (∼10^7^ parasites/mL) were diluted in M199, seeded in 50 µL per well using FlexDrop™ and incubated at 28°C for 28 hours, followed by the addition of 5 mM resazurin sodium salt (Sigma R7017, United States) and further incubation for 20 hours at 28°C. After a 48-hour exposure to compounds, the reference drug (EtBr) or control (1% DMSO), the parasites were fixed with 2% paraformaldehyde (PFA) and plates were read in Victor3™ (Perkin Elmer) at 530 nm (excitation) and 590 nm (emission). This fixation step is not necessary for resazurin readout, but allows flexibility in the automation schedule and increases assay robustness by decreasing metabolic variability between populations across wells and plates. Z-factor, calculated as 1−(3×σ*p*+3×σ*n*)/(|μ*p*−μ*n*|), where μ*p*, σ*p*, μ*n* and σ*n* are the means (μ) and standard deviations (σ) of both the positive (*p*) and negative (*n*) controls [15], and other parameters, including DRC plates for verification of the reference drug EC_50_ (accepted if within the range of 3× higher or lower than a defined value from the literature), coefficient of variation not higher than 10% in the controls and edge effect evaluation, were used for screening validation and hits selection.

### Secondary Screening Assay: Human Cell Toxicity Test

To assess the cytotoxicity of compounds on THP-1, an acute monocytic leukemia-derived human cell line (ATCC TIB-202™), a viability assay also using resazurin reduction with minor modifications in concentration and incubation time was performed. Z-factor [15] and other parameters [16] were used for secondary screening validation and hit exclusion.

### Macrophage Infection and Intracellular Amastigotes Assay for Hit Confirmation

THP-1 cells were cultivated in suspension at a density of 10^5^ to 10^6^ cells/mL in RPMI (Gibco 1640, United States) medium supplemented with 10% heat-inactivated FBS and 1% streptomycin/penicillin at 37°C and 5% CO_2_. The differentiation of THP-1 into macrophages was induced by incubation of the cells for 48 hours with phorbol 12-myristate 13-acetate (PMA) at 50 ng/ml. For infection, late-stage *L. major* or *L. donovani* rich in metacyclic promastigotes was added to differentiated THP-1 cells (ratio of 50 parasites to 1 host cell) in Evotec™ 384-well microplates using a FlexDrop™ (Perkin Elmer) to dispense 50 µL/well. Compounds were added 24 hours after infection and microplates were incubated at 37°C and 5% CO_2_ for 4 days. Amphotericin B as the reference compound (positive control) at 10 µM as the EC_100_ and 1% DMSO (negative control) were used in all the plates for data normalization (see bellow). After 4 days of incubation, remaining free promastigotes were removed by washing five times with PBS and cells were simultaneously fixed with 2% PFA and the DNA stained with 5 µM of Draq5 (Biostatus DR50200, England). Microplates were read in an Opera confocal microscope (Perkin Elmer), enabling the determination of the infection ratio of the parasites by image analysis. An algorithm was developed to identify and individualize the macrophages by setting an intensity threshold to discriminate background (extracellular space) from foreground (macrophage cells area). The macrophage nuclei and internalized parasites were identified and counted if localized in the foreground previously selected (Figure S1). With this technique, extracellular parasites were not considered in the calculations, and the infection ratio was determined by the number of infected cells divided by the total number of cells after normalization based on the controls. The average infection ratios from the positive and negative controls were normalized to 0% and 100% infection, and the infection ratio read from each compound activity was proportionally distributed within this range. Z-factor [15] was used for protocol validation and active compound selection acceptance.

### Microsomal Stability Assay

Compounds at a concentration of 2 µM in 0.2% DMSO were incubated with 0.5 mg/mL rat (Sprague-Dawley Rat, BD Gentest) and human (human pool donors, BD Gentest) liver microsomes in potassium phosphate buffer in a reaction started by the addition of NADPH and stopped either immediately or at 5, 10, 30, 60 or 120 minutes for a precise estimate of microsomal stability. The corresponding loss of the parent compound was determined by a quadrupole liquid chromatography-mass spectrometry (LC-MS) with diode-array detection (Agilent 1200, Agilent Technology). The samples were passed through trapping cartridges (Security Guard Cartridge, Gemini C18, 4×2.0 mm, 3 µm, Phenomenex) followed by an analytical column (Gemini C18, 50×2.0 mm, 3 µm, Phenomenex). Positive electrospray ionization (ESI+) was employed for this analysis. The mobile phases A (water with 0.1% formic acid) and B (acetonitrile with 0.1% formic acid) were used at a flow rate of 0.3 mL/min. Gradient elution started with 95% mobile phase A and 5% mobile phase B. Elution was changed to a linear gradient until 50% A and B for 1 min. This condition was held for 0.5 min, then increased to 95% B over 0.5 min, and held for 1.5 min. Then, the elution gradient returned to 95% A and 5% B over 0.5 min, was held for the remaining 3.5 min. The percentage of the remaining compound was calculated by comparison with the initial quantity at 0 min. Half-life was calculated based on first-order reaction kinetics.

### CYP450 Inhibition Assays

Individual fluorescent probe substrates were used with individual rhCYP isozymes and fluorescence detection according to a previously published method [17]. Probe substrates were 7-benzyloxy-4-(trifluoromethyl)-coumarin (BFC) for CYP3A4 and 3-[2-(*N*,*N*-diethyl-*N*-methylammonium)ethyl]-7-methoxy-4-methylcoumarin (AMMC) for CYP2D6 in 0.5% DMSO. IC_50_ was determined using an eight-point concentration curve with three-fold serial dilutions. Victor3™ (Perkin Elmer) was used for quantification in the fluorescent method (Perkin Elmer Life and Analytical Sciences).

## Results

### HTS Assay Development and Validation

**Figure 1:**
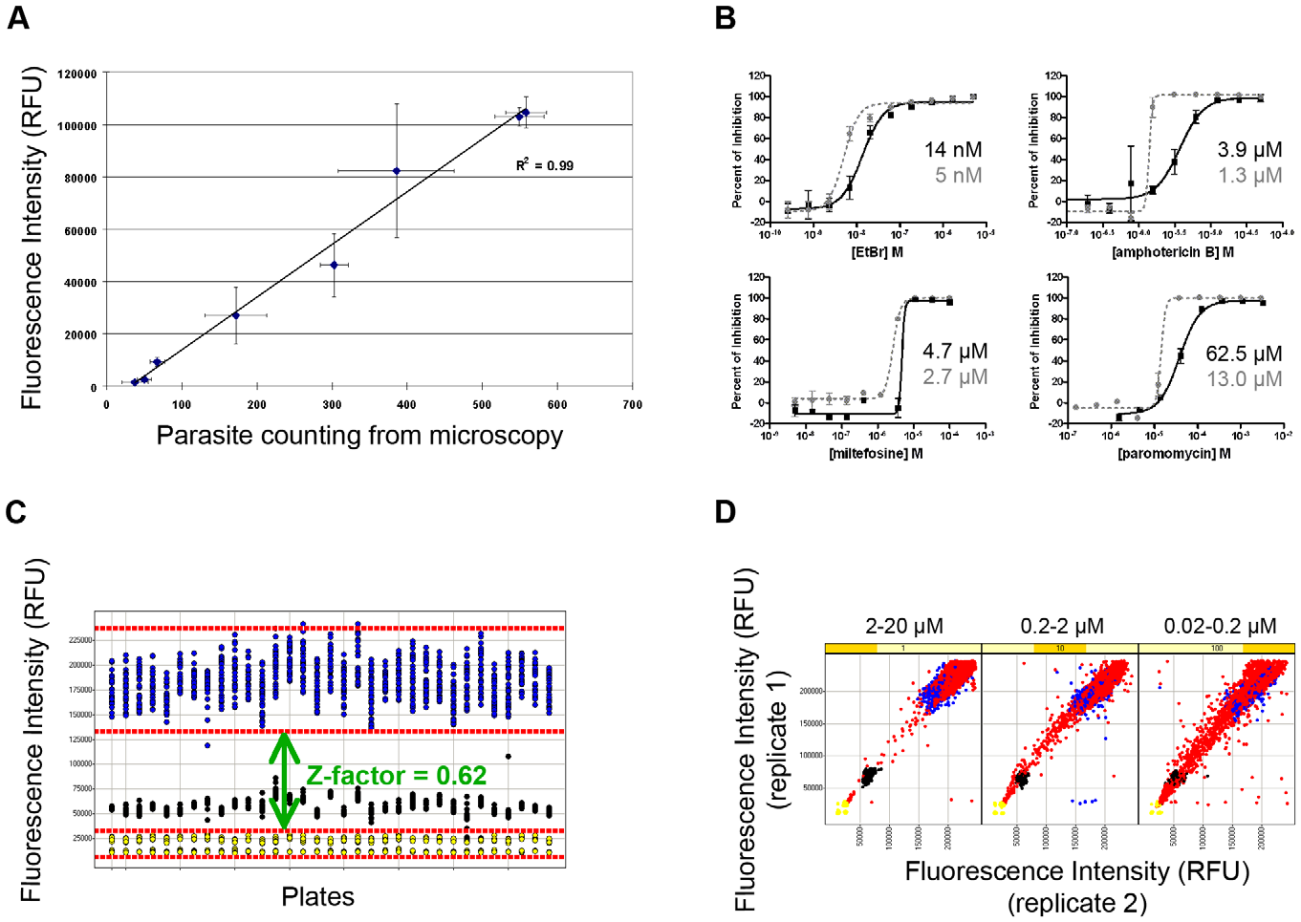
HTS assay validation. **A**) Linear correlation between relative fluorescence unit (RFU) readings from resazurin reduction (y-axis) and parasite number from microscopy counting (x-axis). **B**) Dose response curve and EC_50_ (black line for *L. major* and grey dashed line for *L. donovani*) of the reference compounds used as controls: EtBr, amphotericin B, miltefosine and paromomycin. **C**) Distribution of 33 control microplates showing 1% DMSO as the negative control (blue dots), EtBr EC_50_-30 nM (black dots) and EtBr EC_100_-10 µM as positive controls (yellow dots) and the Z-factor of 0.62. **D**) Distribution plot of the duplicate assay screen of 4,000 compounds (red dots) and controls in three different concentrations, following the same color standards as in C.

The effect of compounds on *Leishmania* viability was assessed by fluorometric measurement of resazurin reduction [13]. Equivalence between the fluorescence signal from resazurin reduction and the number of parasites was confirmed by the linear correlation between parasites counted by light microscopy and the relative fluorescence unit (RFU) value measured (Fig. 1A). Reference compounds for antileishmanial activity (EtBr, amphotericin B, miltefosine and paromomycin) were tested (Fig. 1B) as a step of the validation process. Values obtained for these compounds in our assay in HTS format were similar to those classically reported for these drugs [18], [19]. Assay validation was performed on three separate days using 33 microplates (384 wells/plate) containing only controls. Variability between well-to-well, plate-to-plate and day-to-day were measured to confirm assay robustness, resulting in a Z-factor of 0.62 (Fig. 1C). After assay validation, we screened a 4,000-compound library containing a number of compounds with known pharmacological properties. The screen was performed using three different concentrations with 10× dilution factors for each compound with the highest concentration in the range of 2–20 µM, as compounds in the library did not all have the same molarity. This assay was done in duplicate and results of each concentration assay are represented as individual graphs in Fig. 1D. A 70% proliferation inhibition of the parasites from the lowest compound concentration (0.2–0.02 µM) was the cut-off used for active compound selection. The list of active chemicals included, but was not limited to, previously reported antileishmanial compounds: anisomycin [20], pentamidine isethionate [21], berberine chloride [22], parthenolide [23], nitrofural [24], furazolidone [24] and nifurtimox [25] (data not shown). These results were considered a pharmacological validation of the assay, confirming the ability of the assay to identify antileishmanial compounds.

### HTS

**Figure 2:**
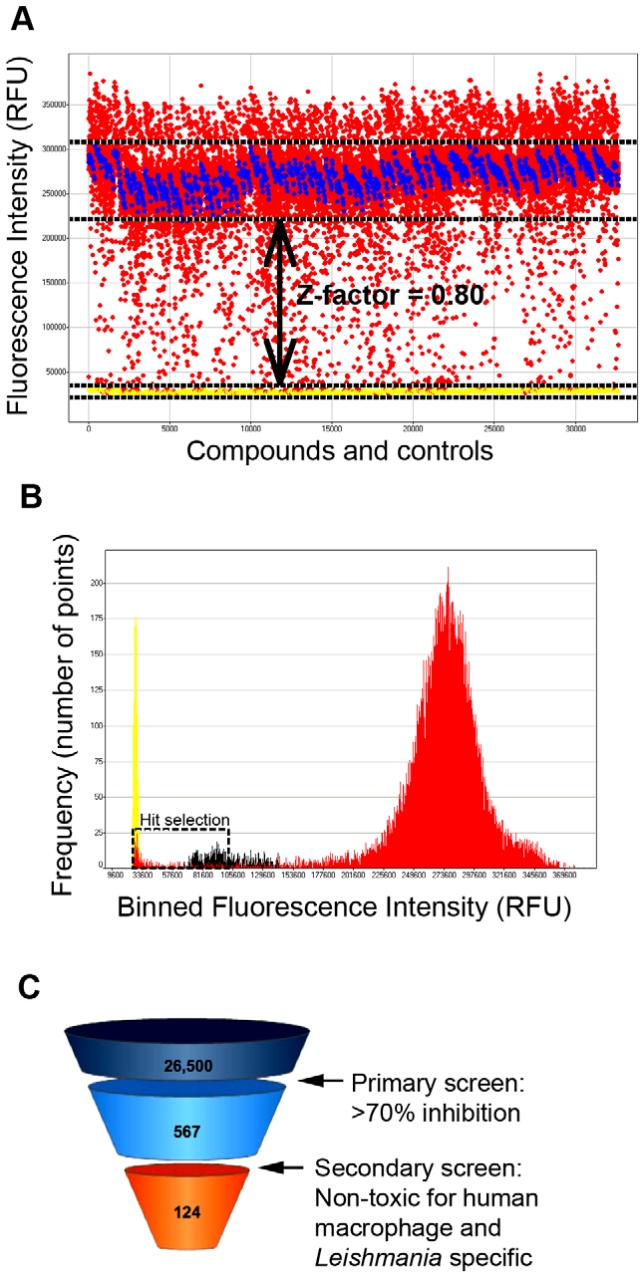
Antileishmanial HTS with 26,500 compounds. **A**) Distribution plot of the 26,500 compounds (red dots), 1% DMSO (blue dots), EC_100_ (yellow dots) and Z-factor = 0.80. **B**) Frequency distribution of the 26,500 compounds based on binned RFUs, highlighting active compound selection with a black dashed box. Data for reference compound EC_100_ in yellow, for reference compound EC_50_ in black and for the 26,500 compounds in red. **C**) A funnel representing selection of antileishmanial activity from 26,500 compounds to 124 hit compounds after primary screening (antileishmanial activity against promastigotes), secondary screening (toxicity exclusion) and no redundancy against *Mycobacterium tuberculosis* or HIV screenings done in-house.

A library containing 26,500 structurally diverse chemical compounds was screened at 10 µM against *L. major*. The positive (EC_100_) and negative controls (1% DMSO) provided a Z-factor of 0.80 (Fig. 2A). The 70% growth inhibition cut-off criterion was used to select the most active compounds. The frequency map distribution based on binned RFUs highlights the distinction of two groups of compounds, in which ∼97% were in the non-active group with RFUs higher than 201,600. Another group contained ∼2% (567 compounds) with RFUs lower than 105,500, representing the active compounds (Fig. 2B). The remaining ∼1% were situated between the other 2 groups and were not sufficiently active for selection.

To exclude potentially toxic compounds from the active list, we performed a secondary viability screening using the non-differentiated human macrophage cell line THP-1. Compounds that interfered with THP-1 viability at a concentration of 10 µM or lower were discarded. In addition, compounds that were found to be active in other in-house, anti-infective HTS campaigns were also excluded to avoid non-specific mechanisms of action. This cytotoxicity determination and *Leishmania* specificity filter resulted in a list of 124 active compounds (Fig. 2C).

### Antileishmanial Activity Confirmation on Intracellular Amastigotes

Infection of human macrophage cells *in vitro* with *Leishmania* has been previously described as a suitable model for screening [26] and was adopted for our purpose. The 124 active compounds selected from the secondary assay were tested in 10-point dose response with 2-fold serial dilutions starting from 20 µM against promastigotes and intracellular amastigotes of *L. major* and *L. donovani* infecting macrophages. The average Z-factor calculated per plate based on the reference drug (amphotericin B) and carrier (1% DMSO) was 0.62 for *L. major* infection and 0.59 for *L. donovani* infection, and minimum accepted Z-factor for the analysis was 0.5. The amphotericin B EC_50_ for *L. major* infecting macrophages was 1.06 µM and EC_50_ for *L. donovani* infecting macrophages was 0.82 µM. From the tested compounds, 5 exhibited activity against *L. major* intracellular amastigotes and are presented in Table 1.

**Table 1:** Active compounds against intracellular *L.major*.

	*L. major*		*L. donovani*		Cytotoxicity CC_50_ THP-1
Compound code	EC_50_ promastigote	EC_50_ amastigote	EC_50_ promastigote	EC_50_ amastigote	
CH872	0.1 µM	0.3 µM	0.05 µM	0.06 µM	>10.0 µM
CA272	2.7 µM	0.8 µM	2.7 µM	19.0 µM	>10.0 µM
NJ231	0.8 µM	11.0 µM	0.6 µM	>20.0 µM	>10.0 µM
CG170	1.0 µM	8.0 µM	0.1 µM	14.0 µM	>10.0 µM
TE122	7.8 µM	12.0 µM	3.0 µM	≫20.0 µM	>10.0 µM

Activity confirmation was based on elimination or growth inhibition of both promastigotes and intracellular amastigotes (parasite forms) of *L. major* and *L. donovani*. Differences between promastigote and intracellular amastigote forms regarding compound susceptibility were demonstrated, as shown in Figure S2. Based on the methods applied, for both *L. major* and *L. donovani* all the compounds, except CA272 for *L. major*, showed more potency against the extracellular form of the parasite (Figure S2).

When comparing species sensitivity we found that some compounds caused different responses in different species. TE122, for example, was only active against the intracellular *L. major* but showed no activity against intracellular *L. donovani* up to 20 µM, as shown in Table 1 and Figure S2. This different drug sensitivity within *Leishmania* species is already known [27] and must be considered in future therapy development.

**Figure 3:**
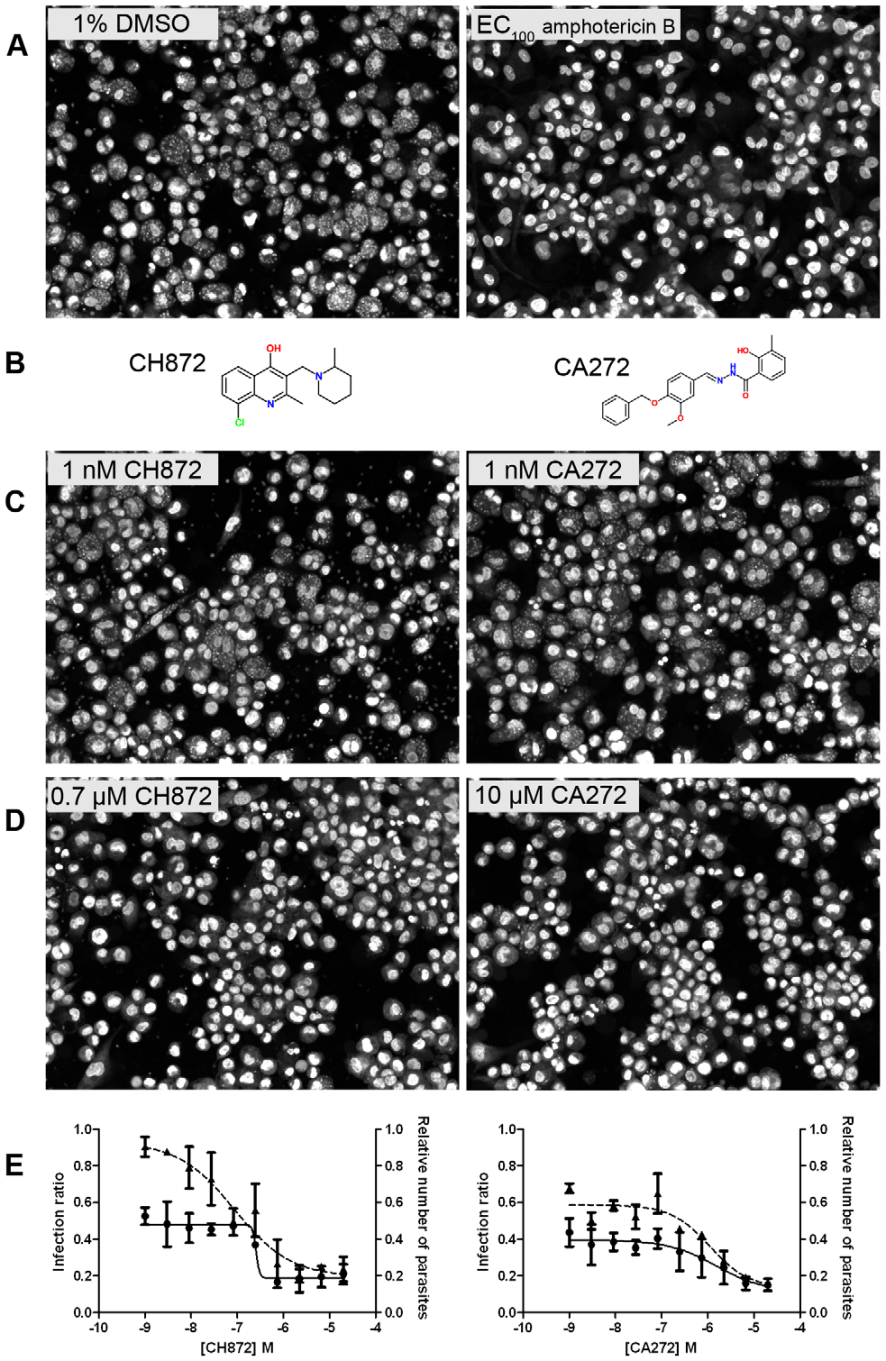
CH872 and CA272 antileishmanial activity against intracellular *L. major* amastigotes. **A**) Infected THP-1 cells in the presence of 1% DMSO as a negative control (left) and EC_100_ obtained with 10 µM of amphotericin B as a positive control (right). **B**) Structures of CH872 (left) and CA272 (right). **C**) Infected THP-1 cells in the presence of 1 nM of CH872 (left) and 1 nM of CA272 (right) as non-active concentrations of the compounds and **D**) in the presence of 0.7 µM of CH872 (left) and 10 µM of CA272 (right) as effective concentrations from the dose-response curves. **E**) Dose-response curves of the compounds CH872 and CA272 plotting the infection ratio (continuous lines) and relative number of parasites compared to the DMSO control (dashed lines).

Using controls with carrier (1% DMSO) and 10 µM amphotericin B (EC_100_ concentration) (Fig. 3A), the two most active compounds, CH872 and CA272 (Fig. 3B), were selected based on image analysis (for details, see Figure S1). These compounds showed significant reduction of macrophage infection with *L. major* after four days incubation (Fig. 3D, E). Moreover, after phenotypic evaluation of the macrophages post-treatment, the compound activity was not due to toxic effects on host cells. Figs. 3C and 3D show, respectively, the infection exposed to a sub-optimal concentration (1 nM) and one example of active concentrations of the compounds (0.7 µM for CH872 and 10 µM for and CA272) according to infection reduction. The dose-response curves against intracellular *L. major* of both compounds plotted in Fig. 3E resulted in a calculated EC_50_ of 0.3 µM for compound CH872 and 0.8 µM for CA272. Only these compounds showed EC_50_s against intracellular *L. major* lower than 1 µM and a ratio CC_50_/EC_50_ (*L. major*) greater than 10 and were selected for further characterization.

### *In vitro* Metabolic Stability Assays

**Figure 4:**
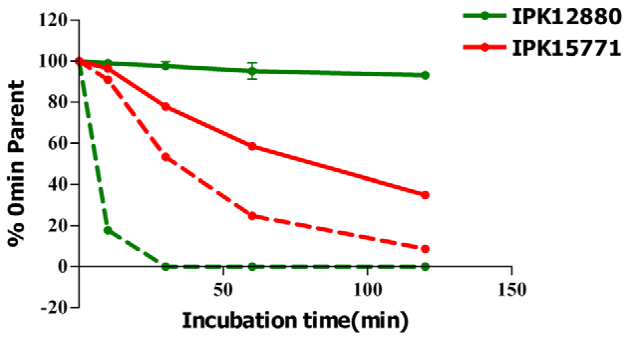
CH872 and CA272 stability in the presence of liver microsomes. Disappearance of CH872 and CA272 in the presence of human (lines) and rat (dashed lines) liver microsomes.

The metabolic stability of the selected compounds in the presence of human and rat liver microsomes was assessed. Compound CH872 was stable in the presence of human liver microsomes, with 99.6% of the parent compound remaining after 30 minutes and a long theoretical half-life. However, it was unstable in rat liver microsomes, being completely degraded after 25 minutes, giving a theoretical half-life of 4.1 minutes (Fig. 4). For CA272, 77.8% of the parent compound remained after 30 minutes in the presence of human liver microsomes, with a theoretical half-life of 79.4 minutes, whereas 53.3% of the parent compound remained in the presence of rat liver microsomes, with a theoretical half-life of 31.8 minutes (Fig. 4). The species difference is most likely due to different enzyme compositions of the liver microsomes. These differences will have to be taken into account if these compounds are to be used with *in vivo* pharmacokinetic models, which are typically performed in rats.

### *In vitro* CYP Inhibition Assays

CYP is a large superfamily of enzymes involved in numerous biological processes [28]. Member of the families CYP2 and CYP3 have a role in drug and steroid metabolism. Toxic side effects as well as drug-drug interactions may be predicted by testing the *in vitro* inhibition of these CYPs by chemical compounds [29]. CYP3A4 and CYP2D6 inhibition assays were performed with both compounds. Whereas CH872 did not inhibit CYP3A4 (EC_50_≫10 µM) or CYP2D6 (EC_50_>10 µM), CA272 did inhibit CYP3A4 with an EC_50_ of 3.41 µM, but did not inhibit CYP2D6 (EC_50_≫10 µM), as shown in Table 2.

**Table 2:** Determination of IC_50_ (µM) of individual human P450s for lead compounds.

Compound	CYP3A4	CYP2D6
CH872	>10	>10
CA272	3.41	>10
Ketoconazole	0.05	–
Quinidine	–	0.02

## Discussion

A high-throughput screening assay for the identification of antileishmanial compounds was developed involving a two-step strategy: primary screening using promastigotes in a 384-well plate format and secondary screening of active compounds using intracellular amastigotes. The primary screening was validated on a robust statistical basis (Fig. 1C) and was demonstrated to be able to identify known antileishmanial compounds when a library of known bioactive small molecules was screened as a proof of principle. This assay was then applied to a screen of 26,500 structurally diverse small molecules. In this screen, 2.1% of the compounds (567) inhibited parasite growth by at least 70% after 48 hours of compound exposure. From these active compounds, almost 80% were excluded due to cytotoxicity or lack of specificity (data not shown), resulting in 124 compounds that were tested against the amastigote in an infection assay with a human macrophage cell line. Although the clinically relevant stage of the *Leishmania* parasites is the intracellular amastigote, the extracellular promastigote poses the obvious advantage of being easier and cheaper to handle in the large scale required by HTS. Besides, promastigotes and amastigotes share common metabolic machinery and pathways, and targets against the first form could be relevant against the second one. This screening strategy against promastigotes was applied by St. George et al. to screen 15,000 compounds against *L. tarentolae* [30] and recently by Sharlow et al. to investigate 200,000 unique compounds for *L. major* growth inhibition [31]. As discussed by the authors of the latter study, the use of the promastigote stage for antileishmanial drug discovery may compromise the discovery of macrophage-metabolized prodrugs, such as antimonials. In the present study, antileishmanial activity of the primary selected compounds was further confirmed against intracellular *L. donovani* amastigotes in a cellular image-based assay in which host cell integrity was taken into account. Although this strategy does not compensate for the possibility of missing prodrugs during the primary screening, it does guarantee that the active compounds are able to cross the macrophage membrane and kill the amastigotes inside the host cells.

As expected, most of the compounds were less active or not active against the intracellular amastigote form when compared to the promastigote, as shown in Table 1 and Figure S2. To be active against the amastigote, a compound must cross two membrane barriers (cellular membrane of the macrophage and phagolysosome vacuole membrane) and maintain stability under low pH and in the presence of free radicals in the phagolysosome environment, which increase the attrition rate compared to the promastigote assay. However, in the promastigote extracellular assay, the parasite is directly exposed to the compound. Furthermore, the concentrations at which compounds show activity do not have an observed effect on the macrophage host cell, confirmed by both a cytotoxicity test and image analysis.

One of the two most active hit compounds was the hydrazine CA272, a novel scaffold for antileishmanial compounds. It exhibited good efficacy in *L. major* infection reduction, although the activity against the intracellular *L. donovani* amastigote was lower. Other unfavorable properties, such as low metabolic stability against human liver microsomes (Fig. 4) and inhibition of CYP3A4 (Table 2), might be improved by the optimization of the two phenyl rings, which can be easily modified. Tests against other *Leishmania* species should also be considered for further studies. Satisfactory activity against intracellular *L. major*, in addition to low toxicity, indicates a good starting point for a new antileishmanial candidate drug.

**Figure 5:**
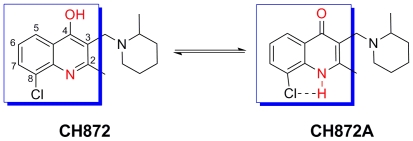
New quinoline with antileishmanial activity. Unlike other quinoline derivatives reported as antileishmanial compounds, CH872 contains a 4-OH group that allows this compound to equilibrate with its tautomer (CH872A). 8-Cl can facilitate the tautomerization by making a H-bond with the NH in CH872A.

The most active compound, CH872, is of interest due to its high *in vitro* activity and lack of cytotoxicity (Fig. 3, Table 1 and Figure S2), along with favorable metabolic stability and CYP inhibition data (Table 2). Additionally, its core structure, 4-hydroxyquinoline, is a novel scaffold for antileshmanial inhibition. Several modifications of quinolines have been carried out to obtain antileishmanials, such as sitamaquine or quinoline derivatives with side-chains at C4 or at phenyl rings [32], [33]; however, none of these modifications produced 4-hydroxyquinoline derivatives. Sitamaquine is in clinical trials (Phase II), and no other quinoline derivatives have been approved as antileishmanial drugs. Some issues reported from this class of compounds include kidney toxicity, which can be lethal [34]. Unlike other antileishmanial quinoline derivatives, compound CH872 contains a 4-OH group, which allows this compound to equilibrate with its tautomer CH872A (Fig. 5). Studies directed toward establishing the structure-activity relationships (SARs) and defining the mode of action of CH872 are currently underway.

The identification of these two antileishmanial compounds demonstrates that primary screening against the promastigote form can be used to identify novel scaffolds that can serve as the starting point for hit-to-lead programs. Using the experience gained in this effort, we are currently developing and implementing an automated high-throughput drug screening assay in 384-well plate format using amastigotes infecting human macrophages as a primary screening assay.

In summary, high-throughput screening of small molecule libraries against promastigotes in a rapid and simple 384-well plate format assay is able to identify novel antileishmanial compounds. Furthermore, these novel compounds have been shown to be active against the intracellular amastigote. As the promastigote screening is much easier and cheaper compared to screening the intracellular amastigote, this strategy could be used to screen libraries of natural compounds commonly available in endemic areas, where resources are often limited. This opens new opportunities for the discovery of future candidates for drug development to be performed locally in endemic countries.

## Supporting Information

Figure S1 Macrophage infection detection: software interface. THP-1 macrophages infected with *L. donovani* amastigotes were fixed and stained with Draq5, and pictures were acquired in the Opera confocal platform as described in the Methods section. A) Raw images (left) are analyzed by the algorithm, which will attribute colors masks (right) to highlight elements detected during the analysis: THP-1 cytoplasm in blue, THP-1 nuclei in red and parasites in green. B) Zoomed detail of the area marked in a red square in (A) showing a non-infected and an infected THP-1 cell before (left) and after (right) the algorithm has been applied to identify intra- and extracellular parasites Note that all parasites identified by the software are highlighted with a cross (+). Green arrows show examples of intra- and extracellular parasites that are, respectively, counted and not counted for infection ratio calculations.
(1.85 MB TIF)

Figure S2 Dose Response Curves (DRCs) of the 24 most active compounds against *L. major* promastigotes. The first 5 compounds (CH872, CA272, NJ231, CG170 and TE122) are presented in Table 1. Black curves refer to activity against promastigotes and blue curves refer to activity against intracellular amastigotes. Continuous black or blue curves refer to *L. major* while dashed curves refer to *L. donovani*. Antileishmanial compound activity is shown on the left Y axis after normalization relative to negative control (0% activity - no drug exposure) and positive control (100% activity - EC100 of the reference drug). The red curves represent the number of THP-1 cells shown in the right Y axis, demonstrating no cytotoxicity effect up to 10 µM of compound exposure (maximal concentration of compound compatible with the assay format).
(0.47 MB TIF)
