# Supplementary figures and images for: Antileishmanial High-Throughput Drug Screening Reveals Drug Candidates with New Scaffolds
Source: PLoS Negl Trop Dis. 2010 May 4;4(5):e675. doi: 10.1371/journal.pntd.0000675 (PMC2864270; doi:10.1371/journal.pntd.0000675)

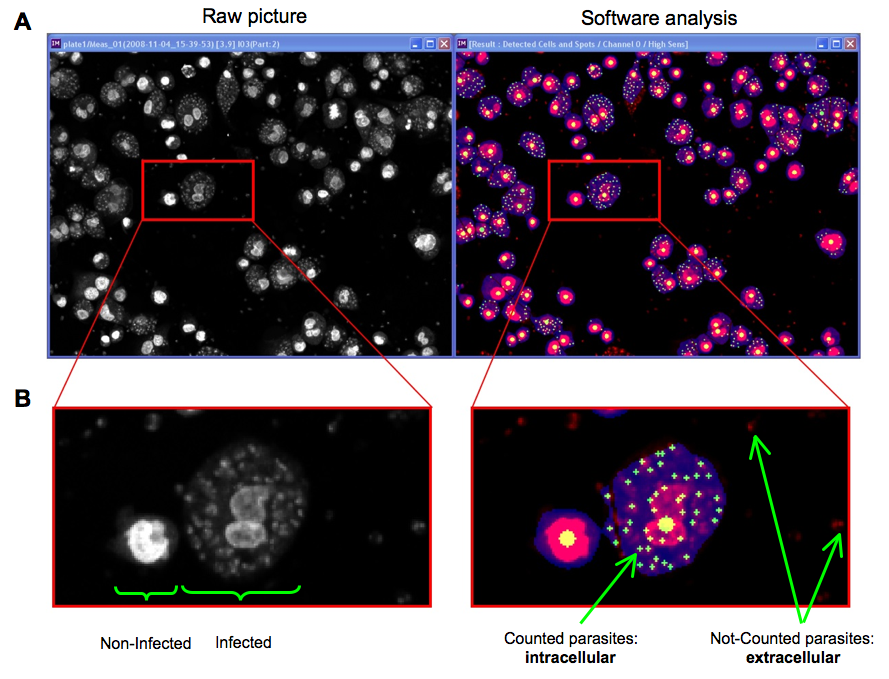

Supplement: Figure S1 — Macrophage infection detection: software interface. THP-1 macrophages infected with L. donovani amastigotes were fixed and stained with Draq5, and pictures were acquired in the Opera confocal platform as described in the Methods section. A) Raw images (left) are analyzed by the algorithm, which will attribute colors masks (right) to highlight elements detected during the analysis: THP-1 cytoplasm in blue, THP-1 nuclei in red and parasites in green. B) Zoomed detail of the area marked in a red square in (A) showing a non-infected and an infected THP-1 cell before (left) and after (right) the algorithm has been applied to identify intra- and extracellular parasites Note that all parasites identified by the software are highlighted with a cross (+). Green arrows show examples of intra- and extracellular parasites that are, respectively, counted and not counted for infection ratio calculations. (1.85 MB TIF) [file pntd.0000675.s001.tif]
